# Diversity and distribution of fish in the Qilian Mountain Basin

**DOI:** 10.3897/BDJ.10.e85992

**Published:** 2022-08-12

**Authors:** Zhaosong Chen, Lijing Luo, Ziwang Wang, Dekui He, Lixun Zhang

**Affiliations:** 1 School of Life Sciences, Lanzhou University, Lanzhou, China School of Life Sciences, Lanzhou University Lanzhou China; 2 Yuzhong Mountain Ecosystems Observation and Research Station, Lanzhou University, Lanzhou, China Yuzhong Mountain Ecosystems Observation and Research Station, Lanzhou University Lanzhou China; 3 Institute of Hydrobiology, Chinese Academy of Sciences, Wuhan, China Institute of Hydrobiology, Chinese Academy of Sciences Wuhan China; 4 University of Chinese Academy of Sciences, Beijing, China University of Chinese Academy of Sciences Beijing China; 5 College of Ecology, Lanzhou University, Lanzhou, China College of Ecology, Lanzhou University Lanzhou China

**Keywords:** Qilian Mountain drainage basins, diversity inventory, spatial pattern, historical connectivity

## Abstract

The Qilian Mountain Basin, on the north-eastern edge of the Qinghai-Tibet Plateau (QTP), supports a high diversity of native and endemic fish. However, the detailed species inventory and distribution patterns concerning fish in the whole Basin remain unknown, which hinders the conservation of biodiversity and assessment of ecological health. We compiled a comprehensive species richness and distribution database of freshwater fish in the Qilian Mountain Basin, based on field investigations and exhaustive data collection from 50 rivers or lakes. Then, we elucidated a distribution pattern using clustering and ordination analyses based on a β_dissim_ matrix with species presence/absence data. A total of 79 freshwater fish species within eight orders, 17 families and 42 genera were recorded. The Qilian Mountain Basin could be grouped into six systems, which match the six Basins (i.e. Heihe River Basin, HHR; Qaidam Basin, QDM; Qinghai Lake Basin, QHL; Shule River Basin, SLR; Shiyang River Basin, SYR; Yellow River Basin, YR), based on the fish distribution pattern. Additionally, the spatial pattern of species distribution showed the distance decay of taxonomic similarity. Our results demonstrate that riverine connectivity resulting from historical processes plays a vital role in shaping the freshwater ichthyofauna of High Central Asia. These findings will be valuable for future systematic conservation of fish in the Qilian Mountain Basin.

## Introduction

Identifying regional species pools and obtaining distribution records play important roles in both understanding the significance of biogeographical processes (e.g. speciation, evolution and dispersal) in shaping biotas and providing valuable information for policy-makers and managers to develop effective protection strategies (Brook et al. 2008, Mendoza-Ponce et al. 2020). In the era of the sixth mass extinction of species, organisms in freshwater ecosystems are more significantly threatened than those in terrestrial and marine ecosystems (Collen et al. 2014, Bongaarts 2019). Freshwater fish have received widespread attention as the most imperilled taxa in freshwater ecosystems, whose distributions are subject to multiple stressors, including anthropogenic interference, water conservancy construction and biological invasion (Olden et al. 2010, Carrizo et al. 2013). Thus, freshwater fish diversity research and conservation have become more imminent and important than ever before (Chen et al. 2021).

The Qilian Mountain Basin, located on the northeast slope of the QTP in northwest China, is an essential ecological safety shelter in western China and a priority area for biodiversity conservation in China (Liu et al. 2003, Qian et al. 2019). The composition of the ichthyofauna in the Qilian Mountain Basin shows a high degree of unity with the QTP, with Schizothoracinae (Cypriniformes, Cyprinidae) and *Triplophysa* (Cypriniformes, Nemacheilidae) as the dominant species (He et al. 2020). In recent decades, however, the natural habitat of native fish has been destroyed due to anthropogenic activities, such as cascade hydropower development, alien species invasion and water resources overexploitation; hence, the riverine communities and fish diversity of the Qilian Mountain Basin have also changed (Feng et al. 2017, Tang et al. 2021). Studies have been performed to investigate fish diversity and threat factors, but these studies were generally focused on local areas or tributaries (Tang et al. 2013, Wang et al. 2015). In addition, previous studies on fish diversity were mostly based on fieldwork, whereas very few of them involved the compilation of comprehensive historical data. As a result, knowledge of the status of stocks throughout the Basin remains unclear.

Mountains play an important role in regional riverine ecosystems (Viviroli et al. 2007, Anderson and Goulden 2011) and the formation of their biota is more sensitive than that of other areas to geological history (Tang et al. 2009, He et al. 2022). Mountain ecosystems, such as the QTP, characterised by the largest quantity of glaciers and known as the water tower of Asia, are the regions of origin and maintenance of a substantial fraction of rivers and plateau lakes (Immerzeel et al. 2020). Unlike that of terrestrial groups, the dispersal of freshwater species is limited by water systems and scarcely across mountain barriers (Rahel 2007). Therefore, analyses of the spatial pattern of freshwater fish can identify some biogeographical signals that might not be detected in terrestrial organisms (Leroy et al. 2019). The rise of the QTP since the Pliocene has strongly affected the evolution of the surrounding river systems. Originating in stages during the uplift of the QTP and affected by its dynamic geological process (e.g. glacial-interglacial cycles) (Li 1963, Li et al. 2001), the water systems of the Qilian Mountain Basin experienced repeated connection and separation events. For example, the Qaidam River system, Qinghai Lake and Shiyang River originally drained into the Yellow River, but the connection was severed and turned the drainage inwards, forming the endorheic basin after an uplift of the QTP (Feng 1981a, Chen 1988, Li et al. 2000). Several studies on native fish have been conducted to identify these biogeographic signals, but these studies were generally focused on local areas or single taxa (O’Bryan et al. 2010, Zhao et al. 2011). Therefore, whether the imprint that riverine connectivity resulting from historical processes plays a vital role in shaping the freshwater ichthyofauna of High Central Asia (He et al. 2020) can be captured throughout the Qilian Mountain Basin based on all indigenous species datasets, remains unknown.

To fill these various knowledge gaps, we coded the diversity of fish in the Qilian Mountain Basin. The spatial pattern of fish throughout the Basin was described in detail. The results of our research will contribute to the overall fish dataset and have important implications for future systematic conservation of the Qilian Mountain Basin. Moreover, our study provides primary data for the conservation of biodiversity and assessment of ecological health of the QTP.

## Material and methods

### Study area

**Figure 1.**
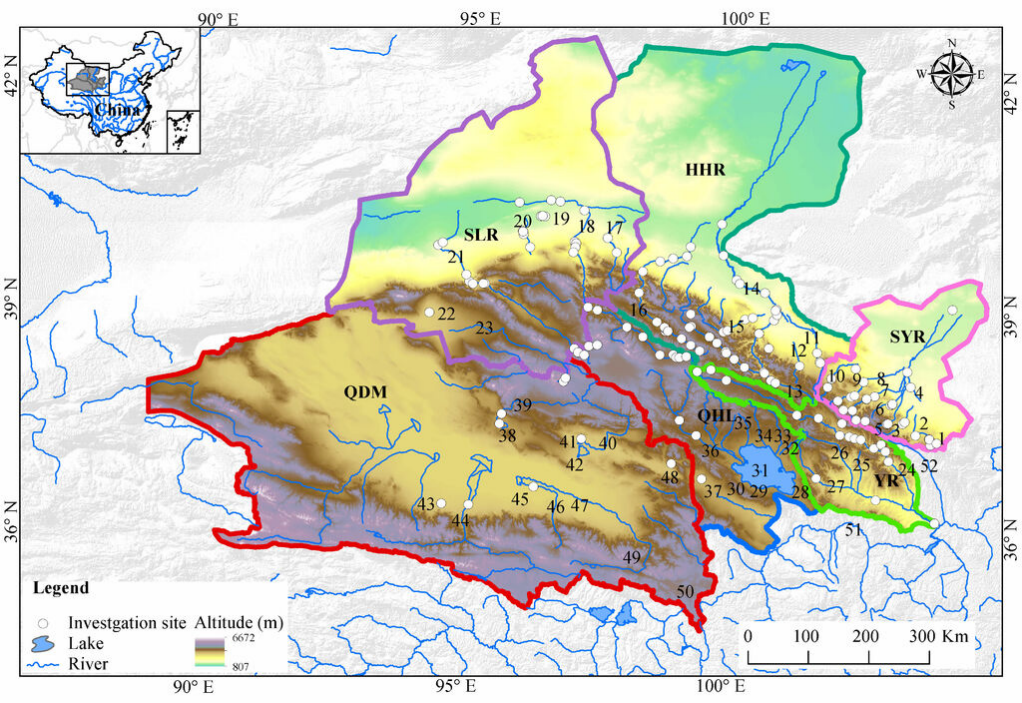
Map of the study area. Basin codes: HHR, Heihe River Basin; QDM, Qaidam Basin; QHL, Qinghai Lake Basin; SLR, Shule River Basin; SYR, Shiyang River Basin; YR, Yellow River Basin. Six Basins' boundaries are outlined with curves of different colours: HHR with dark green, QDM with red, QHL with green, SLR with purple, SYR with pink, YR with green. River codes: 1, Dajing River (DJR); 2, Gulang River (GLR); 3, Huangyang River (HYR); 4, Hongshui River (HSR1); 5, Zamu River (ZMR); 6, Shiyang River (SYR1); 7, Xiying River (XYR); 8, Dongda River (DDR); 9, Jingchuan River (JCR); 10, Xida River (XDR); 11, Damaying River (DMYR); 12, Hongshuida River (HSDR); 13, Babao River (BBR); 14, Heihe River (HHR1); 15, Liyuan River (LYR); 16, Beida River (BDR); 17, Shiyou River (SYR2); 18, Shule River (SLR1); 19, Lucao River (LCR); 20, Yulin River (YLR); 21, Danghe River (DHR); 22, Sugan Lake (SGL); 23, Dahaerteng River (DHETR); 24, Datong River (DTR); 25, Shatangchuan River (STCR); 26, Beichuan River (BCR); 27, Huangshui River (HSR); 28, Daotang River (DTR1); 29, Zhihaique River (ZHQR); 30, Heima River (HMR); 31, Qinghai Lake (QHL1); 32, Ganzi River (GZR); 33, Haergai River (HEGR); 34, Quanji River (QJR); 35 Shaliu River (SLR2); 36, Buha River (BHR); 37CKYL, Chakayan Lake; 38, Chaidan Lake (CDL); 39, Tataleng River (TTLR); 40, Bayin River (BYR); 41, Keluke Lake (KLKL); 42, Tuosu Lake (TSL); 43, Tuolahai River (TLHR); 44, Geermu River (GEMR); 45, Nuer River (NER); 46, Nuomuhong River (NMHR); 47, Qaidam River (QDMR); 48, Dulan River (DLR); 49, Xiangride River (XRDR); 50, Tuosuo Lake (TSL1); 51, Yellow River; 52, Zhuanglang River.

The Qilian Mountain Basin lies in the arid and semi-arid region of north-western China, on the north-eastern edge of the QTP. The study area has a catchment basin of approximately 1,100,000 km^2^ on the north-eastern margin of the QTP spanning an elevation range from 807 to 6,672 m (Fig. 1). Rivers around the Qilian Mountain Basin flow through Gansu, Inner Mongolia and Qinghai Provinces in China and display a radial distribution (Fig. 1). Geographically, the central region is located at the headwaters of the Beida River (Number 16, see Fig. 1) and Buha River (Number 36, see Fig. 1), closest to the QHL, the largest inland saltwater lake in China. The three main endorheic drainage systems, which are derived from the north-eastern part of the Qilian Mountain Basin, are the Shiyang (Number 6, see Fig. 1), Heihe (Number 14, see Fig. 1) and Shule (Number 18, see Fig. 1) Rivers from east to west, which flow north into the Hexi Corridor and finally disappear into the desert. On the southwest side of the Qilian Mountain Basin is the QDM endorheic river system. To the east of the Qilian Mountain Basin, the Huangshui River (Number 27, see Fig. 1) and Datong River (Number 24, see Fig. 1) drain into the Yellow River (Number 51, see Fig. 1) system, the only outflow river basin of the Qilian Mountain Basin.

### Fish dataset

A fish species taxonomic checklist was constructed from 88 sources of published literature (82 journal articles and six theses) and 11 books (Suppl. material 1). Species and their distribution data were further supplemented with specimens deposited in the Museum of Lanzhou University and records from our five field surveys conducted at 148 sites across the Qilian Mountain Basin from 2019 to 2021 (sampling sites as shown in Fig. 1). All fish species were identified, based on their morphological characteristics and the specimens that were difficult to identify, especially for *Triplophysa* , were verified using molecular sequencing (detailed methods as previously described by Chen et al. 2021). Valid species names were invoked as found in Eschmeyer’s Catalog of Fishes (Fricke et al. 2022).

**Figure 2.**
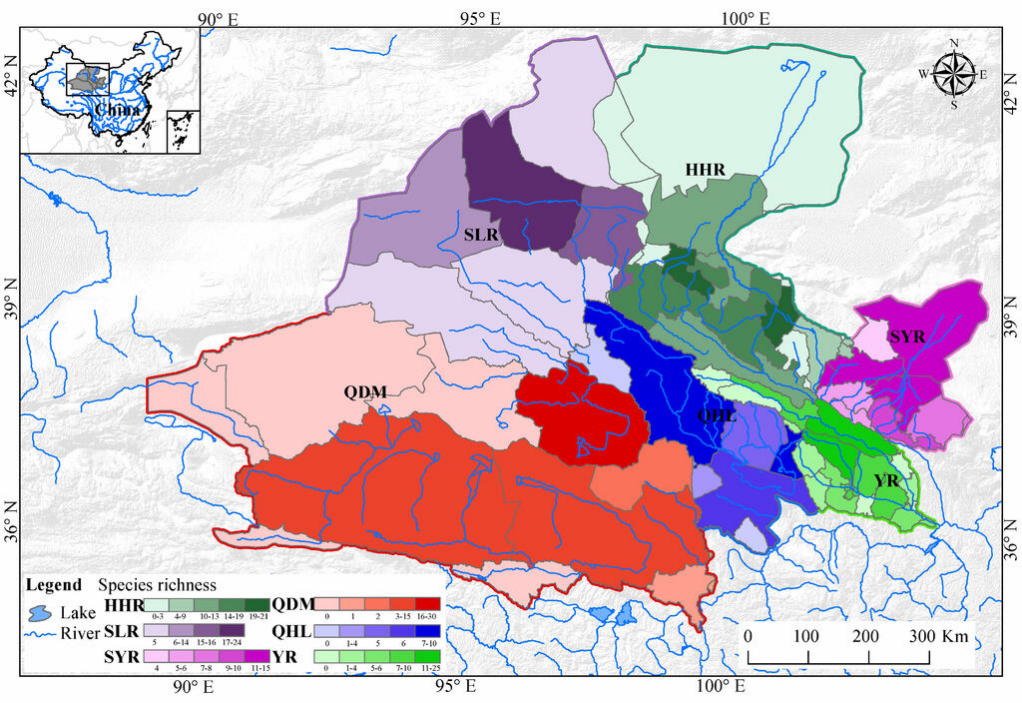
The species richness pattern of fish in the Qilian Mountain Basin. Basin codes are shown in Fig. 1.

The Qilian Mountain Basin was classified into 51 county-level hydrologic units depending on the accuracy of the site locality in the past and the county area (Fig. 2, Suppl. material 2) using ArcGIS 10.7 software. Non-native species were included in our study and defined in terms of historical documents (Yang and Zhang 1991, Li et al. 2009, Li and Kang 2012, Tang and He 2015). Then, the fish distribution points with presence/absence data were marked and grouped into each river or lake and hydrologic unit.

To identify the conservation status of native fish in the Qilian Mountain Basin, we recognised if the species were at risk of extinction using the Red List of China’s Vertebrates (Jiang et al. 2016). Fish species assessed as Critically Endangered (CR), Endangered (EN) or Vulnerable (VU) are referred to as “threatened” in this study. We also assigned the species to protection class categories, including at the national (https://www.forestry.gov.cn/) and provincial levels (Zhang and Zhao 2016).

### Statistical analysis

Diversity was quantified by species richness, which is the total number of fish species of each unit and river or lake. The spatial pattern of species richness, based on county-level hydrological units, was presented. Additionally, we visualised the co-occurrence of native, exotic and endemic species across the six Basins. Given the ability of fish species to disperse along the river network, we took 50 rivers or lakes (Number 1-50, see Fig. 1) in the Qilian Mountain Basin as river-level units, based on presence/absence data of native species to increase the comparability amongst them. The native species distribution β_dissim_ matrix was derived using the Bray-Curtis coefficient to describe the relationships amongst all the units (Holt et al. 2013). Then, the group average linkage cluster model was performed to map the fish faunistic relationships of the Qilian Mountain Basin. We used non-metric multidimensional scaling (NMDS; Kruskal 1964) ordination to illustrate faunistic dissimilarity. We calculated stress values to assess the fit between the NMDS results and the original dissimilarity matrix. This index falls between 0 and 1, with values lower than 0.2 indicating credible NMDS results.

We used a one-way analysis of similarity (ANOSIM; Clarke and Warwick 1994) to assess compositional differences in the native fish assemblage. The statistic *R* ranges from -1 to 1, with values close to 1 indicating high separation amongst groups in community composition. To identify indicator taxa and better understand the ichthyofauna characteristics of each region, we used the linear discriminant analysis (LDA) effect size (LEfSe) method to capture significant differences in ichthyofauna amongst regions (with LDA scores > 3; Segata et al. 2011). Indicator species analysis (ISA; Dufrêne and Legendre 1997) was used to identify the species that contributed most to the delineation of regions. Species with ISA values > 0.5 were considered as the indicator. In addition, we used the similarity percentages (SIMPER; Clarke 1993) algorithm to identify the species that contributed most to similarities amongst groups detected by the NMDS.

The map of China (including county-level administrative boundaries, national boundaries and rivers) used in this study was obtained from the National Geomatics Center of China (http://www.ngcc.cn/ngcc/). The programme PRIMER Version 7 (Clarke and Warwick 2001) was used for the group average linkage cluster analysis. The LEfSe technique was performed using Wekemo Bioincloud tools, a free online platform for data analysis (https://www.bioincloud.tech). All other analyses were performed in the R Version 4.0.3 environment (R Core Team 2020) using the “ggplot2”, “indicspecies”, “permute”, “tidyverse”, “vegan” and “UpsetR” packages.

## Results

### Species composition

A total of 79 freshwater fish belonging to eight orders, 17 families and 42 genera were found in the study area (Suppl. material 3). Cypriniformes (59 species) and Nemacheilidae (26 species) were the most species-rich order and family, respectively. At the genus level, *Triplophysa* accounted for 31.6% (25 species) of the total, followed by *Gymnocypris* (four species, 5.1%) and *Rhinogobius* (four species, 5.1%). Amongst the rivers or lakes (Table 1), the Huangshui and Heihe Rivers had the highest richness (41 species), followed by the Datong River and Keluke Lake (28 species), Beichuan River (21 species), Beida River (20 species) and Shule River (20 species). Amongst the six Basins (Fig. 2, Table 2), the YR contained a total of 49 species, ranking first, followed by the HHR (43 species), QDM (33 species), SLR (30 species) and SYR (29 species). The QHL concerned the lowest fish biodiversity at 12 species.

**Figure 3.**
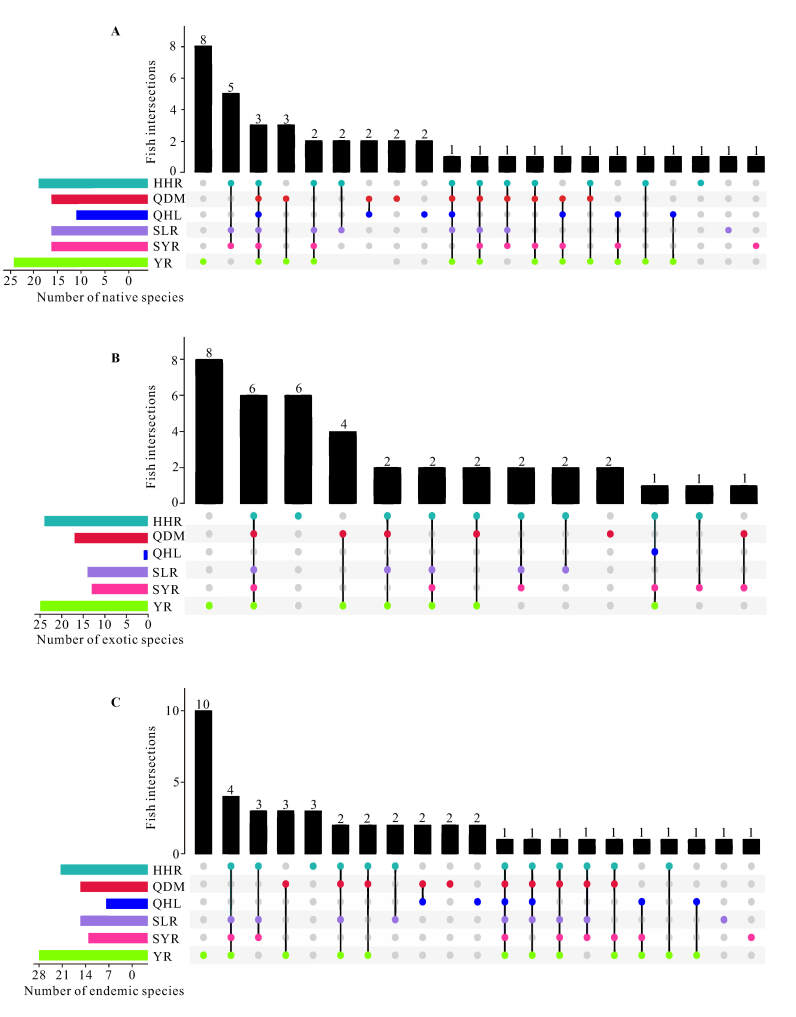
Distribution of **A** native, **B** exotic and **C** endemic fish species across six Basins in the Qilian Mountain Basin; codes are shown in Fig. 1. Vertical bars show the number of species occurring in each of the unique combinations of basin represented by the single point for a set of species unique to one basin and the connected points for a set of species co-occurring in multiple basins (e.g. **A** five native fish species occurred in the HHR, SLR and SYR, but in no other basins).

At the same time, there were 40 native and 38 exotic fish species in the Qilian Mountain Basin (Suppl. material 3). Notably, *Carassiusauratus* is a native species in the Hexi River Basin, but also an exotic species in the other three Basins. The YR had the highest native (24 species) and exotic (25 species) species richness, followed by the HHR (19 species and 24 species, respectively). The QHL hosted only one non-native species (Table 2, Fig. 3A and B). The species richness of exotic fish found in the SLR and SYR was more than half that of native fish and even exceeded that of native fish in the HHR, QDM and YR (Table 2). We found that the highest number of native species in common was recorded amongst the HHR, SLR and SYR, followed by the YR and QDM (Fig. 3A). We also found that the YR, QDM and QHL had more native species recorded exclusively in each Basin than the SLR, HHR and SYR (Fig. 3A). In terms of exotic species composition, we detected a higher number of co-existing species amongst the five Basins other than the QHL (Fig. 3B). Some non-native species, such as *Misgurnusanguillicaudatus*, *Paramisgurnusdabryanus*, *Cyprinusrubrofuscus*, *Abbottinarivularis*, *Pseudorasboraparva*, *Opsariichthysbidens* and *Micropercopsswinhonis*, have widely established feral stocks in diverse aquatic ecosystems (e.g. wetlands, rivers and reservoirs) of each Basin.

We identified 45 species endemic to China in the Qilian Mountain Basin (Suppl. material 3). The Basin with the most endemic species was the YR (28 species), followed by the HHR (22 species), QDM (16 species), SLR (16 species) and SYR (13 species). The QHL hosted the fewest endemic species (eight species) (Table 2, Fig. 3C). Based on endemic species composition, we found similar results for co-existing native species: the HHR, SLR and SYR, as well as the YR and QDM, had more endemic species in common (Fig. 3C).

The conservation status of the fish species in the Qilian Mountain Basin is listed in Suppl. material 4. Amongst the 40 native fish species, *Triplophysacakaensis* was classified as Extinct (EX) and 37.5% (15 species) were categorised as threatened (i.e. CR, EN and VU). *Chuanchialabiosa*, *Gymnodiptychuspachycheilus*, *Platypharodonextremus* and *Triplophysasiluroides* were listed as national class II protected wildlife (Suppl. material 4). A total of 13 fish species were classified as aquatic wildlife with provincial class key protection (Suppl. material 4). Therein, four species (*P.extremus*, *Leuciscuschuanchicus*, *T.siluroides* and *Siluruslanzhouensis*) were key protected species in both Gansu and Qinghai Provinces. These threatened and protected fish species mainly appear in the rivers of the YR (Tables 1, 2).

### Spatial pattern

**Figure 4.**
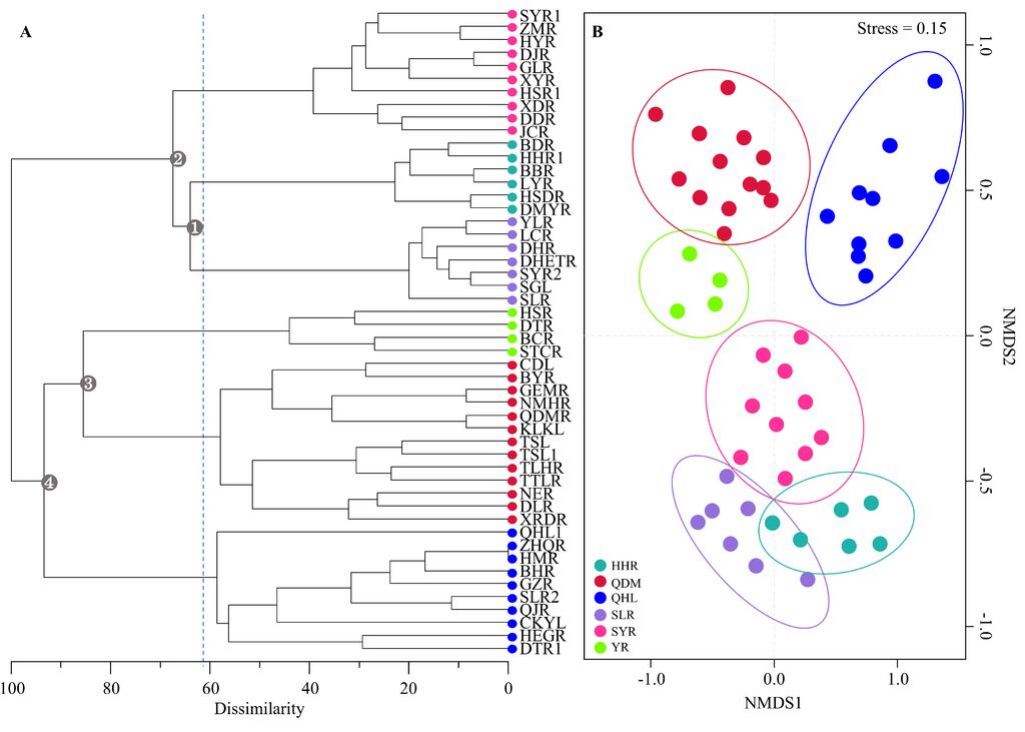
**A** Cluster analysis of 50 rivers or lakes fish data for the Qilian Mountain Basin, based on the Bray-Curtis dissimilarity matrix and group average clustering method. **B** Non-metric multidimensional scaling analysis of freshwater fish in 50 rivers or lakes of the Qilian Mountain Basin, based on the Bray-Curtis dissimilarity matrix. Basin and river codes are shown in Fig. 1.

The cluster analysis results for 50 rivers or lakes, based on the Bray-Curtis coefficient, showed that the Qilian Mountain Basin could be divided into six groups when the β_dissim_ index was approximately 62 (Fig. 4A). The water bodies on the six cluster branches corresponded exactly to the six water systems of the Qilian Mountain Basin. The HHR first merged with the SLR at a β_dissim_ value of approximately 65 (Node 1, see Fig. 4A) and then clustered with the SYR at a β_dissim_ value of approximately 68 (Node 2, see Fig. 4A). The YR was first merged with the QDM at a β_dissim_ value of approximately 85 (Node 3, see Fig. 4A) and then clustered with the QHL at a β_dissim_ value of approximately 93 (Node 4, see Fig. 4A). Finally, these two groups merged and clustered (Fig. 4A). Geographically, the water bodies at Nodes 2 and 4 in the clustering dendrogram were merged into the northern (Gansu area) and southern (Qinghai area) regions of the Qilian Mountain Basin, respectively (Figs 1, 4A). In addition, the geographically adjacent rivers or basins were clustered into one group (Figs 1, 4A), indicating that the fish community structure presented significant spatial autocorrelation. The six groups yielded by clustering analysis were also clearly separated from each other in the two-dimensional ordination space of NMDS (Stress = 0.15; Fig. 4B). ANOSIM also demonstrated good agreement between the cluster assignments (Global test *R* = 0.512, *P* = 0.001).

**Figure 5.**
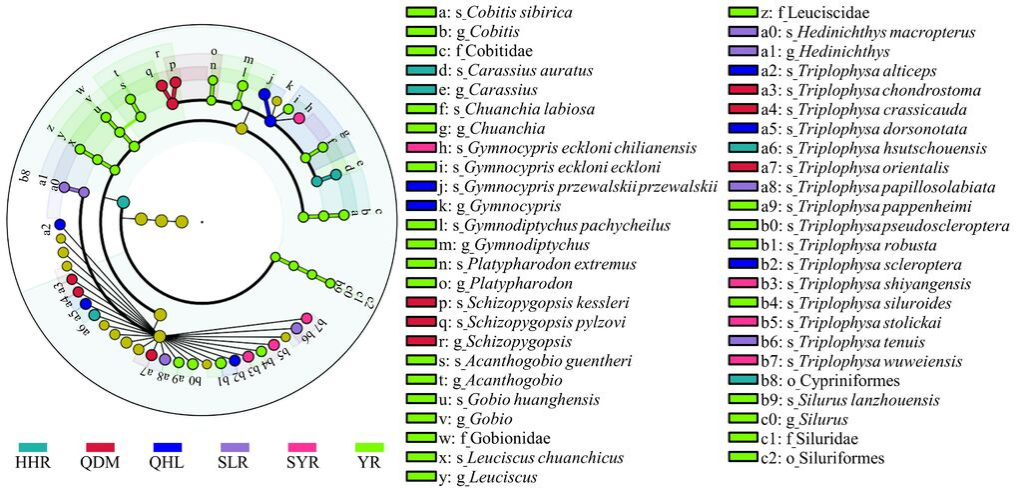
The cladogram of six Basins, based on native species data at four taxonomic categories (i.e. order, family, genus and species). Basin codes are shown in Fig. 1.

Six Basins were characterised by different taxa in the LEfSe and ISA results (Fig. 5, Table 2, Suppl. material 5). The species that contributed most to indicator species were endemic or dominant species in each watershed, consistent with the results identified through the LEfSe technique (Fig. 5, Table 2, Suppl. material 5). The QDM, SLR, SYR and QHL were featured as rich in the genera *Gymnocypris*, *Schizopygopsis* and *Triplophysa* (Fig. 5). The HHR was dominated by *C.auratus* and *Triplophysahsutschouensis* (Fig. 5). The YR was distinguished from the other Basins by its outstanding characteristics with *Cobitissibirica*, *C.labiosa*, *G.pachycheilus*, *P.extremus*, *Acanthogobioguentheri*, *Gobiohuanghensis*, *L.chuanchicus* and *S.lanzhouensis.* Additionally, in the SIMPER analysis, the average similarity amongst Basins ranged from 27.9% to 57.8% (Table 2). Moreover, 81.0% overall average dissimilarity amongst regional species compositions was observed (Table 2). The dissimilarity was mostly attributable to the species *T.hsutschouensis* (12.0%) and *Gymnocyprisprzewalskiiprzewalskii* (11.3%).

## Discussion

**Diversity characteristics**

We first coded the regional fish species pools for the Qilian Mountain Basin with the most extensive database of freshwater fish distributions for 79 species, 42 genera, 17 families and eight orders. The Qilian Mountain Basin, with a complex water system and abundant biodiversity, is an important vertebrate aggregation and glacial refuge on the QTP (Qu et al. 2005, Zhang et al. 2005, Qi et al. 2007, Zhao et al. 2007, Zhao et al. 2011). The fish species composition of the Qilian Mountain Basin presents typical QTP ichthyofauna, with Schizothoracinae and *Triplophysa* mainly residing on the QTP (Wu and Wu 1992). The north-eastern edge of the QTP contains a high number of plateau loach species. A total of 25 *Triplophysa* species were recorded in this study, which is greater than the number from field surveys of the north-eastern QTP (22 species in Wang et al. 2020) and accounts for approximately 75.8% of the total number of plateau loach species on the QTP and its adjacent regions (33 species in Li et al. 2017). Of these species, 80% (20 species) are endemic to China. The species richness of the subfamily Schizothoracinae in the Qilian Mountain Basin was high (nine species, 11.4% of total species) and their morphological diversity showed two of three specialisation levels of the subfamily (Cao et al. 1981). According to our study, one species of *Gymnodiptychus* belongs to the specialised grade (i.e. the whole body scales are moderately or entirely degenerated) and one species of *Chuanchia*, four species of *Gymnocypris*, one species of *Platypharodon* and one species of *Schizopygopsis* belong to the highly specialised grade (i.e. the whole body scales are entirely degenerated). No species belongs to the primitive grade (i.e. the whole body is covered by scales or with moderate degeneration). These results indicated that fish of the Qilian Mountain Basin are important model species for biodiversity conservation and ecological evolution research on the QTP.

In general, fish diversity is closely related to the geographical environment, drainage area and fish evolutionary history of rivers (MacArthur and Wilson 1967, Tang et al. 2009). The YR of the Qilian Mountain is characterised by the highest regional runoff, high habitat heterogeneity and primary productivity due to its upstream region of the YR (Zhao et al. 2020). In addition, there was also a relatively stable climate environment in the geological history period, which was especially less affected during the uplift of the QTP and Quaternary glacial advance (Wu and Wu 1987, Zhang et al. 2003). Both contribute to the YR hosting the highest fish diversity in the Qilian Mountain Basin (49 species, 62.0% of total species). Furthermore, the YR is characterised by high endemic and threatened fish diversity, which is also in the demand for ecological protection. The QDM possesses the largest basin area with the highest number of rivers amongst the six Basins in the Qilian Mountain and, thus, provides more habitat and plays a positive role in fish diversity (Báldi 2008). Due to the extreme conditions (e.g. high pH and salinity; Zhu and Wu 1975, Zheng 1997), combined with decreased rainfall, only a few fish species reside in the QHL (12 species, 15.2% of total species). The geographical environment (e.g. precipitation and temperature) of the Hexi River system becomes less suitable from east to west along the Qilian Mountain Basin. This is also evidenced by the genetic diversity in the population of *Triplophysaleptosoma* gradually decreasing from east to west due to the geographical environment of the three Basins (Zhang et al. 2017). In combination with the drainage area (Table 2), this resulted in fewer fish in the SYR, followed by the SLR and HHR.

Characterised by high levels of native fish biodiversity and endemism, the Qilian Mountain Basin is very important for fish biodiversity conservation. Despite its high diversity, nearly half of the non-native freshwater fish species occurred in the Qilian Mountain Basin. Native species play a vital role in biodiversity conservation and dominate the ecosystem function (Risser 1995). Special attention should be given to the future development of indigenous fish under the pressure of invasive alien species.

**Spatial distribution**

The ichthyofaunal composition of the Qilian Mountain Basin was distinctively divided and corresponded to the six Basins: the HHR, QDM, QHL, SLR, SYR and YR (Fig. 4). Our results for the Qilian Mountain Basin also provide empirical evidence that historical riverine connectivity plays a vital role in shaping the spatial pattern of freshwater fish in High Central Asia by facilitating exchanges between populations. Historically, the Shiyang River, Qinghai Lake and Qaidam endorheic river system originally drained into the Yellow River, but the connection was successively severed from east to west with the interval uplift of the QTP (Feng 1981a, Chen 1988, Li et al. 2000), which may cause the difference in the similarities of fish faunal composition between each of the three Basins and the YR due to freshwater fish genetic exchange demanded with the river network. The clustering and NMDS ordination results, based on β_dissim_, captured the imprint of this historical process. The ichthyofauna of the YR most closely resembled that of the QDM, followed by the QHL and SYR. Species co-occurrence data also support this pattern (Fig. 3).

Stratigraphic evidence shows that the headwater area of the YR existed in the QDM as a series of lakes in the early stage, so the YR was historically connected with the QDM (Zhang et al. 2003). This can explain that most fish (approximately 68.8%; Suppl. material 3) were shared by the QDM and YR in the Qilian Mountain. This finding is also supported by several phylogenetic and biogeographical studies of Schizothorax (Qi et al. 2007, Zhao et al. 2007, Qi et al. 2015) and *Triplophysa* (Zhang 2017, Zhang et al. 2017), which indicated that the populations from the QDM have a very close phylogenetic relationship with those from the YR. The ancient Qinghai Lake (Number 31, see Fig. 1) originally drained to the southeast and was linked with the Yellow River (Number 51, see Fig. 1) and the connection was severed because of the uplift of the Mountains (Chen 1988). When these findings are considered with our results, there is no doubt that the existing fish fauna of the QHL should be homologous to that of the YR. This finding is in accordance with the results of a phylogeographical study of *G.przewalskiiprzewalskii* by Zhao et al. (2007), who demonstrated that *G.przewalskiiprzewalskii* of the QHL originated from *Gymnocypriseckloniecklon* of the YR. The Gulang River (Number 2, see Fig. 1) of the SYR originally drained to the southeast and connected with the Zhuanglang River (Number 52, see Fig. 1) of the YR and the connection was severed after the uplift of the Qilian Mountain Basin (Feng 1981a). This evidence is supported by the typical co-occurrence of species (i.e. *Schizopygopsispylzovi*, an endemic species to the YR) and *G.ecklonichilianensis*, the closest relatives of *G.ecklonieckloni* (Zhao 1986), which were recorded in both Basins in this study. More fish species were shared by the HHR and SLR, which lends support to the evidence that the Heihe River (Number 14, see Fig. 1) connected with the Shule River (Number 18, see Fig. 1) in the ancient period (Feng 1981a). This finding is in line with phylogenetic studies of *Triplophysa* showing that the populations (e.g. *T.leptosoma*) from the HHR and SLR are closely related (Zhang 2017, Zhang et al. 2017). Our finding of close geographic affinities in fish populations from SYR, HHR and SLR is supported by a phylogeographic study of one endemic species, *G.ecklonichilianensis*, by Zhao et al. (2011), who found that the SYR is likely where *G.ecklonichilianensis* first appeared, after which it gradually expanded westwards along the HHR and SLR. Additionally, this finding is consistent with species co-occurrence data (Fig. 3). Furthermore, there is evidence that the Shule River (Number 18, see Fig. 1) once flowed westwards into Lop Nor and connected with the Tarim River water system (Feng 1981b). In terms of current distributions, several loach fish are also shared by the Shule River and Tarim River water systems, such as *Hedinichthysyarkandensis* and *Triplophysatenuis* (Chen et al. 2017). This study also showed that adjacent rivers or basins shared higher biotic similarity, which is known as the distance decay of taxonomic similarity (Nekola and White 1999).

The six Basins presented unique ichthyofauna characteristics with differences in dominance or endemicity (Fig. 5, Table 2, Suppl. material 5). Schizothoracinae and *Triplophysa* are typical plateau fish adapted to extreme environmental conditions, such as high altitude, cold water and poor nutrition (Chen et al. 1996). With the different ecological environments and food resources amongst watersheds, the fish are undergoing adaptive differentiation in various traits, such as the degree of scale degradation in Schizothorax (He et al. 2004, He and Chen 2007) and the shape of the air bladder in *Triplophysa* (Feng et al. 2019), gradually speciation in each watershed and even each river of the Qilian Mountain. However, the indicator species *C.sibirica*, *G.pachycheilus*, *A.guentheri*, *G.huanghensis* and *L.chuanchicus* in the YR are distinct from those in the other Basins. One explanation is that these species were isolated in the YR and were less affected by the uplift of the QTP than Schizothorax and *Triplophysa* (Wu and Wu 1987). Another explanation might be that speciation occurred long before the connection between the north-eastern QTP and the Yellow River system. The species evolved and adapted to the plateau water environment along with the connection between the upper reaches of the Yellow River and Longxi Basin in the middle and late Pleistocene (Wu and Wu 1987).

## Limitations

Several limitations in our study deserve to be mentioned. First, our findings are subject to some uncertainty due to sub-standard data, deficient taxonomy and insufficient sampling efforts. Second, our map of the spatial distribution pattern was derived from fish presence/absence data, but without considering the phylogenetic relationships of taxa. Phylogenetic information measures the time-scale of inter-species evolution and the evolutionary relationships between species (Forest et al. 2007). Therefore, our map is expected to be understood at a finer scale by combining the reported data with molecular phylogenetic data. Additionally, despite providing valuable insights into understanding how historical processes play a vital role in shaping the freshwater ichthyofauna of High Central Asia, this study did not cover other mechanisms driving the spatial distribution of species. Area, energy and history hypotheses are generally used to explain the underlying mechanisms shaping the spatial patterns of biodiversity (Field et al. 2009, He et al. 2020). However, limited by the grain of recorded distribution data and poor methodologies, testing these three hypotheses is very challenging.

## Conclusions

In the present study, a list of 79 fish species in the Qilian Mountain Basin was compiled for the first time, based on both field surveys and data collections. Our results clearly mapped the species pool division of the Qilian Mountain Basin, based on the β_dissim_ index. Additionally, the spatial pattern of species distribution showed the distance decay of taxonomic similarity. Therefore, this study captured the imprint that riverine connectivity resulting from historical processes plays a vital role in shaping the freshwater ichthyofauna of High Central Asia. These findings have important implications for the systematic conservation of fish species in the Qilian Mountain Basin and provide primary data for the conservation of biodiversity and assessment of ecological health of the QTP.

## Supplementary Material

68E30AE0-833D-5B0E-889C-423158456B57 10.3897/BDJ.10.e85992.suppl1 Supplementary material 1 Data source of fish distribution information in the Qilian Mountain Basin
Data type references File: oo_676711.pdf https://binary.pensoft.net/file/676711 authors of this paper

800BB8C8-E1BE-5580-9812-7DDB62425C5F 10.3897/BDJ.10.e85992.suppl2 Supplementary material 2 The division of county-level hydrologic units in the Qilian Mountain Basin
Data type image File: oo_716216.jpg https://binary.pensoft.net/file/716216 authors of this paper

336C244F-5306-5650-B5CA-4D71F6B76D0C 10.3897/BDJ.10.e85992.suppl3 Supplementary material 3 The inventory of fish in the Qilian Mountain Basin Data type occurences File: oo_716219.pdf https://binary.pensoft.net/file/716219 authors of this paper

61D25C1B-341F-5C6C-A5EC-EB43279C0C82 10.3897/BDJ.10.e85992.suppl4 Supplementary material 4 Conservation status of native fish species in the Qilian Mountain Basin Data type occurences File: oo_676724.pdf https://binary.pensoft.net/file/676724 authors of this paper

E7D327AC-7409-5F2E-838D-0F1216A89626 10.3897/BDJ.10.e85992.suppl5 Supplementary material 5 Histogram of the LDA scores computed for features differentially abundant amongst six basins
Data type image File: oo_676491.jpg https://binary.pensoft.net/file/676491 authors of this paper

## Figures and Tables

**Table 1.** Fish diversity of main rivers in the Qilian Mountain Basin. River codes are shown in Fig. 1.

**River**	**Total Species**	**Exotic Species**	**Endemic Species**	**Threatened Species**
HSR	41	23	21	9
DTR	28	6	19	9
BCR	21	11	15	5
HHR1	41	23	20	3
BDR	20	9	9	1
SLR1	20	9	11	2
DHR	9	2	5	1
SYR1	19	9	7	1
QHL1	7	0	4	1
BHR	7	0	4	1
KLKL	28	16	12	2
GEMR	16	6	9	2
QDMR	8	0	8	2
NMHR	9	0	9	1

**Table 2.** Characteristics of each Basin in the Qilian Mountain Basin. Basin codes are shown in Fig. 1.

**Basin**	**HHR**	**QDM**	**QHL**	**SLR**	**SYR**	**YR**	**Total**
Area (km^2^)	249,033.07	393,023.21	88,311.63	241,074.98	65,657.88	48,802.89	1,085,903.66
No. of Total Species	43	33	12	30	29	49	79
No. of Native Species	19	16	11	16	16	24	40
No. of Exotic Species	24	17	1	14	13	25	38
No. of Endemic Species	22	16	8	16	13	28	45
No. of Threatened Species	4	3	2	3	2	11	15
No. of Provincial key protected species	3	3	3	1	2	9	13
No. of National key protected species	0	0	0	0	0	4	4
No. of Indicator Species (native species)	2	5	4	3	4	13	31
SIMPER (native species)	44.4%	27.9%	42.3%	40.7%	57.8%	55.0%	81.0%
