# Supplementary material for: Diversity and distribution of fish in the Qilian Mountain Basin
Source: Biodivers Data J. 2022 Aug 12;10:e85992. doi: 10.3897/BDJ.10.e85992 (PMC9848581; doi:10.3897/BDJ.10.e85992)
Supplement: Supplementary material 1 — Data source of fish distribution information in the Qilian Mountain Basin [file bdj-10-e85992-s001.pdf]

References

(a) Journal articles

- Bao XK, Yang ZW, Zhao W, Shi CH, Yang YW, Wang L (2014) The alteration of the vertebrate resources over the past two decades in Gansu Anxi Extreme Arid National Nature Reserve. Biodiversity Science 22 (4): 539-545. [In Chinese].
- Cao WX, Zhu SQ (1988) Two new species of the genus *Triplophysa* from Qinghai-Xizang Plateau, China. Acta Zootaxonomica Sinica 13 (2): 201-204. [In Chinese].
- Chao Y, Shen ZX, Wang GJ, Yang C, Qi DL (2011) Distribution and properties of amylase in alimentary tract and hepatopancreas of *T. siluroides.* Journal of Anhui Agricultural Sciences 39 (23): 14139-14140, 14212. [In Chinese].
- Chen D, Zhang X, Tan X, Wang K, Qiao Y, Chang Y (2009) Hydroacoustic study of spatial and temporal distribution of *Gymnocypris przewalskii* (Kessler, 1876) in Qinghai Lake, China. Environmental Biology of Fishes 84: 231-239. https://doi.org/10.1007/s10641-008-9430-y
- Chen SA, Wang C, Yao N (2013) Biology on *Triplophysa* (*T.*) *siluroides* (Herzenstein) from Beichuanhe Basin of Qinghai Province. Journal of Gansu Agricultural University 48 (4): 34-39. [In Chinese].
- Chen S, Xie C, Li D, Yao N, Ding H, Zhang Z (2017) Length-weight relationships of five *Triplophysa* species from the northwest of China. Journal of Applied Ichthyology 33 (6): 1234-1236. https://doi.org/10.1111/jai.13432
- Du YY, Zhang YP, Yang ZY, Wang T (2021) Length-weight relationships of four fish species from the Hei River in northwest China. Journal of Applied Ichthyology 37 (2): 362-363. https://doi.org/10.1111/jai.14172
- Feng CG, Tang YT, Liu SJ, Tian F, Zhang CF, Zhao K (2019) Multiple convergent events created a nominal widespread species: *Triplophysa stoliczkae* (Steindachner, 1866) (Cobitoidea: Nemacheilidae). BMC Evolutionary Biology 19 (1): 177. https://doi.org/10.1186/s12862-019-1503-3
- Feng CG, Tong C, Zhang RY, Li GG, Wanghe KY, Tang YT, Zhang CF, Zhao K (2017) Biodiversity and distribution patterns of *Triplophysa* species in the northeastern margin of the Tibetan Plateau. Biodiversity Science 25 (1): 53-61. [In Chinese].
- Feng CG, Wu YJ, Tian F, Tong C, Tang YT, Zhang RY, Li GG, Zhao K (2017) Elevational diversity gradients of Tibetan loaches: The relative roles of ecological and evolutionary processes. Ecology and Evolution 7 (23): 9970-9977. https://doi.org/10.1002/ece3.3504
- Feng CG, Zhou WW, Tang YT, Gao Y, Chen JM, Tong C, Liu SJ, Wanghe KY, Zhao K (2019) Molecular systematics of the *Triplophysa robusta* (Cobitoidea) complex: Extensive gene flow in a depauperate lineage. Molecular Phylogenetics and Evolution 132: 275-283. https://doi.org/10.1016/j.ympev.2018.12.009
- Guo XY, Xie L, Zhang XZ, Ji YF, Chen J, Pang B, Xu YT, Qi DL, Guo SC (2016) Signatures of

functional constraint at Fgfr1a Genes in schizothoracine fishes (Pisces: Cypriniformes): The dermal skeleton variation adapted to high-altitude environments. Integrative Zoology 11 (2): 86-97. https://doi.org/10.1111/1749-4877.12178

- He DK, Chen YF (2007) Molecular phylogeny and biogeography of the highly specialized grade schizothoracine fishes (Teleostei: Cyprinidae) inferred from cytochrome *b* sequences. Chinese Science Bulletin 52 (6): 777-788. https://doi.org/10.1007/s11434-007-0123-2
- He DK, Chen YX, Chen YF (2006) The molecular phylogeny and biogeography of genus *Triplophysa*. Progress in Natural Science 16 (11): 1395-1404. [In Chinese].
- He DK, Chen YF, Chen YY, Chen ZM (2004) Molecular phylogeny of the specialized schizothoracine fishes (Teleostei: Cyprinidae), with their implications for the uplift of the Qinghai-Tibetan Plateau. Chinese Science Bulletin 49 (1): 39-48. https://doi.org/10.1007/BF02901741
- Hu HY (2008) Construction of Datong River Shitouxia Hydropower Station and fish protection. Journal of Qinghai Environment 18 (3): 113-116, 122. [In Chinese].
- Lei MT, Cai JZ, Li CH, Fu Y, Sun J, Ma DD, Li YP, Zhang YM (2020) Prevalence and genetic diversity of *Echinorhynchus gymnocyprii* (Acanthocephala: Echinorhynchidae) in schizothoracine fishes (Cyprinidae: Schizothoracinae) in Qinghai-Tibetan Plateau, China. Parasites & Vectors 13 (1): 357. https://doi.org/10.1186/s13071-020-04224-w
- Li GP (2013) Investigation and reflection on fish resources in Datong area. Animals Breeding and Feed 5: 72-74. [In Chinese].
- Li JX, Wang Y, Jin HF, Li WJ, Yan CC, Yan PF, Zhang XY, He SP, Song ZB (2017) Identification of *Triplophysa* species from the Qinghai-Tibetan Plateau (QTP) and its adjacent regions through DNA barcodes. Gene 605: 12-19. https://doi.org/10.1016/j.gene.2016.11.045
- Li KM, Tang WJ, Guan HT (2009) Protection measures for indigenous fish species in Qinghai Province. Journal of Hydroecology 2 (3): 32-36. [In Chinese].
- Li LL, Quan JQ, Wang NL, Zhao GY, Luo ZC, Kang YJ, Liu Z (2021) Analysis of genetic diversity and phylogeny on mtDNA D-loop of *Triplophysa*. Genomics and Applied Biology 40 (1): 143-152. [In Chinese].
- Li QS, Kang PT (2012) General situation of aboriginal fish resources and utilization and protection strategy in Gansu province. Freshwater Fisheries 42 (3): 92-96. [In Chinese].
- Li QS, Shao DH, Qin Y, Kang PT, Gao XY, Tang PW, Wang WL (2013) Investigation report on fishery resources in Gansu section of Heihe River. Gansu Agriculture 5: 17-21. [In Chinese].
- Li SZ, Zhang SY (1974) Two new species and one new subspecies of fishes from the northern part of Kansu Province, China. Current Zoology 20 (4): 414-419. [In Chinese].
- Li WJ, Chen XC, Hu YP (2015) A new species of the genus *Triplophysa* (Nemacheilinae), *Triplophysa qilianensis* sp. nov, from Qinghai, China. Zootaxa 3905 (3): 418-424. https://doi.org/10.11646/zootaxa.3905.3.7

- Lou JM, Yang TY, Wang T, Gao J (2020) The fish community phylogenetic structure and its mechanism of community construction in the three continental river in Gansu Province. Journal of Hubei University (Natural Science) 42 (1): 42-48. [In Chinese].
- Lou JM, Zhang Z, Wang T, Gao J (2019) Genetic diversity and population differentiation of three Schizothoracinae fishes in Gansu Province. Journal of Huazhong Agricultural University 38 (4): 77-84. [In Chinese].
- Lv WQ, Lei Y, Deng Y, Sun N, Liu X, Yang LD, He SP (2020) Accelerated evolution and positive selection of rhodopsin in Tibetan loaches living in high altitude. International Journal of Biological Macromolecules 165: 2598-2606. https://doi.org/10.1016/j.ijbiomac.2020.10.151
- O'Bryan DM, Xie Z, Wang Y, Du J, Brauner CJ, Richards JG, Wood CM, Chen XQ, Murray BW (2010) Phylogeography and conservation genetics of Lake Qinghai scaleless carp *Gymnocypris przewalskii*. Journal of Fish Biology 77 (9): 2072-2092. https://doi.org/10.1111/j.1095-8649.2010.02792.x
- Qi DL, Chao Y, Guo SC, Zhao LY, Li TP, Wei FL, Zhao XQ (2012) Convergent, parallel and correlated evolution of trophic morphologies in the subfamily schizothoracinae from the Qinghai-Tibetan plateau. PloS One 7 (3): e34070. https://doi.org/10.1371/journal.pone.0034070
- Qi DL, Chao Y, Guo SC, Zhao XQ (2008) Genetic structure of five Huanghe schizothoracin *Schizopygopsis pylzovi* populations based on mtDNA control region sequences. Acta Zoologica Sinica 54 (6): 972-980. [In Chinese].
- Qi DL, Chao Y, Liang J, Gao Q, Wu RR, Mather L, Zhao YL, Chen QC (2018) Adaptive evolution of interferon regulatory factors is not correlated with body scale reduction or loss in schizothoracine fish. Fish & Shellfish Immunology 73: 145-151. https://doi.org/10.1016/j.fsi.2017.12.013
- Qi DL, Guo SC, Chao Y, Kong QH, Li CZ, Xia MZ, Xie BS, Zhao K (2015) The biogeography and phylogeny of schizothoracine fishes (*Schizopygopsis*) in the Qinghai-Tibetan Plateau. Zoologica Scripta 44 (5): 523-533. https://doi.org/10.1111/zsc.12116
- Qi DL, Guo SC, Tang WJ, Zhao XQ, Liu JQ (2007) Mitochondrial DNA phylogeny of two morphologically enigmatic fishes in the subfamily Schizothoracinae (Teleostei: Cyprinidae) in the Qinghai-Tibetan Plateau. Journal of Fish Biology 70: 60-74. https://doi.org/10.1111/j.1095-8649.2007.01366.x
- Qi DL, Guo SC, Tang WJ, Yang J, Zhao XQ (2006) Molecular systematics of morphologically similar fishes in the Schizothoracinae in Nanmenxia River, with implication for morphological convergent evolution. Acta Zoologica Sinica 52 (5): 862-870. [In Chinese].
- Qi DL, Guo SC, Zhao XQ (2006) Molecular systematics of two enigmatic fishes in the genus *Schizopygopsis* in the Qinghai-Tibetan Plateau. Acta Zoologica Sinica 52 (6): 1058-1066. [In Chinese].
- Qi DL, Guo SC, Zhao XQ, Yang J, Tang WJ (2007) Genetic diversity and historical population structure of *Schizopygopsis pylzovi* (Teleostei: Cyprinidae) in the Qinghai-Tibetan Plateau. Freshwater Biology 52 (6): 1090-1104. https://doi.org/10.1111/j.1365-2427.2007.01731.x

- Qi DL, Li TP, Chao Y, Yang C (2009) Taxonomic status of *Schizopygopsis kessleri* inferred from Cyt *b* gene sequence. Chinese Journal of Zoology 44 (4): 1-7. [In Chinese].
- Quan JQ, Zhao GY, Li LL, Zhang JP, Luo ZC, Kang YJ, Liu Z (2021) Phylogeny and genetic diversity reveal the influence of Qinghai-Tibet Plateau uplift on the divergence and distribution of *Gymnocypris* species. Aquatic Sciences 83 (1): 1-11. https://doi.org/10.1007/s00027-020-00761-9
- Song WW, Xu Q, Fu XQ, Wang CFZ, Pang Y, Song DH (2019) EFDC simulation of fishway in the Diversion Dahaerteng River to Danghe Reservoir, China. Ecological Indicators 102: 704-715. https://doi.org/10.1016/j.ecolind.2019.03.025
- Tang WJ, Chen YF, Ding CC (2013) The current situation and protection of fish resources in Huangshui River in Qinghai Province. Journal of Dalian Ocean University 28 (3): 307-313. [In Chinese].
- Tang WJ, He DK (2015) Investigation on alien fishes in Qinghai Province, China (2001-2014). Journal of Lake Sciences 27 (3): 502-510. [In Chinese].
- Tang WJ, Shen ZX, Jian SL (2006) Rare and endangered fishes of the Yellow River in Qinghai Province and protection countermeasures. Reservoir Fisheries 26 (1): 57-60. [In Chinese].
- Tang WJ, Wang M, Li KM (2005) Indigen fish in Qinghai Province. Chinese Journal of Fisheries 18 (1): 13-17. [In Chinese].
- Tang WJ, Zhao X, Zhang MT (2012) Investigation on aquatic organisms in the upper stream of Heihe River in Qinghai Province, China. Journal of Dalian Ocean University 27 (5): 477-482. [In Chinese].
- Tang YT, Li CH, Wanghe KY, Feng CG, Tong C, Tian F, Zhao K (2019) Convergent evolution misled taxonomy in schizothoracine fishes (Cypriniformes: Cyprinidae). Molecular Phylogenetics and Evolution 134: 323-337. https://doi.org/10.1016/j.ympev.2019.01.008
- Tang YT, Zhang Y, Zhou BZ, Wang GJ, Jian SL, Li KM, Zhao K (2021) Investigation of fish resources in Qilian Mountains of Qinghai Province. Journal of Gansu Agricultural University 56 (1): 1-7. [In Chinese].
- Tian F, Liu SJ, Shi JQ, Qi HF, Zhao K, Xie BS (2019) Transcriptomic profiling reveals molecular regulation of seasonal reproduction in Tibetan highland fish, *Gymnocypris przewalskii*. BMC Genomics 20 (1): 2. https://doi.org/10.1186/s12864-018-5358-6
- Tong C, Tian F, Zhao K (2017) Genomic signature of highland adaptation in fish: A case study in Tibetan Schizothoracinae species. BMC Genomics 18 (1): 948. https://doi.org/10.1186/s12864-017-4352-8
- Wang T, Qi DS, Sun SH, Liu ZH, Du YR, Guo SC, Ma JB (2019) DNA barcodes and their characteristic diagnostic sites analysis of Schizothoracinae fishes in Qinghai province. Mitochondrial DNA Part A 30 (4): 592-601. https://doi.org/10.1080/24701394.2019.1580273
- Wang T, Zhang YP, Guan LH, Du YY, Lou ZY, Jiao WL (2015) Current freshwater fish resources and the application of DNA barcoding in species identification in Gansu Province. Biodiversity Science 23 (3): 306-313. [In Chinese].

- Wang T, Zhang YP, Yang ZY, Liu Z, Du YY (2020) DNA barcoding reveals cryptic diversity in the underestimated genus *Triplophysa* (Cypriniformes: Cobitidae, Nemacheilinae) from the northeastern Qinghai-Tibet Plateau. BMC Evolutionary Biology 20 (1): 151. https://doi.org/10.1186/s12862-020-01718-0
- Wang Y, Shen YJ, Feng CG, Zhao K, Song ZB, Zhang YP, Yang LD, He SP (2016) Mitogenomic perspectives on the origin of Tibetan loaches and their adaptation to high altitude. Scientific Reports 6: 29690. https://doi.org/10.1038/srep29690
- Wang ZJ, Shen ZX, Wang GJ (2012) A preliminary report on breeding experiment of *Coregonus muksum* in plateau area. Hebei Fisheries (11): 32-34. [In Chinese].
- Wu YF, Tan QJ (1991) Characteristics of the fish-fauna of the characteristics of Qinghai-Xizang Plateau and its geological distribution and formation. Acta Zoologica Sinica 37 (2): 135-152. [In Chinese].
- Wu YF, Wu CZ (1987) Notes on fishes in Huanghe drainage of Qinghai Province, with a faunal analysis. Acta Biologica Plateau Sinica 7: 141-153. [In Chinese].
- Xu GF, Xu LH (1990) Experiment of marsh fish in transplanted pond of Keluke Lake, Qinghai Province. Freshwater Fisheries 6: 33-34. [In Chinese].
- Yang YT, Tang YQ (1995) Resources and geographical distribution of the fishes in Gansu Province, China. Journal of Gansu Sciences 3: 72-75. [In Chinese].
- Yang YT, Zhang YM (1991) Study on fishes' fauna and evolution of the inland river, Hexi Corridor. Journal of Lanzhou University (Natural Sciences) 27 (4): 141-144. [In Chinese].
- Yao N, Ma L, Jin SS, Chen SA (2019) Growth characteristics of *Triplophysa siluroides* (Herzenstein) in Beichuanhe Basin of Qinghai. Journal of Inner Mongolia Agricultural University (Natural Science Edition) 40 (6): 5-10. [In Chinese].
- Zhang CF, Tong C, Ludwig A, Tang YT, Liu SJ, Zhang RY, Feng CG, Li GG, Peng ZG, Zhao K (2018) Adaptive evolution of the *Eda* gene and scales loss in Schizothoracine fishes in response to uplift of the Tibetan Plateau. International Journal of Molecular Sciences 19 (10): 2953. https://doi.org/10.3390/ijms19102953
- Zhang CL, Zhang YL (1963) New species of fish in Qinghai. Acta Zoologica Sinica 15 (4): 635-638. [In Chinese].
- Zhang CL, Zhang YL (1965) Several fishes in lake Zhaling and Datong river of Qinghai province. Chinese Journal of Zoology 3: 121-123. [In Chinese].
- Zhang F, Zhu LN, Zhang LX, Wang WB, Sun GJ (2017) Phylogeography of freshwater fishes of the Qilian Mountains area (*Triplophysa leptosoma*, Cobitidae: Cypriniformes). Environmental Biology of Fishes 100: 1383-1396. https://doi.org/10.1007/s10641-017-0650-x
- Zhang JP, Liu Z, Zhang B, Yin XY, Wang L, Wang JF (2015) MtDNA Cyt *b* and D-loop sequence feature and genetic differentiation of *Gymnocypris chilianensis* and *G. przewalskii*. Freshwater Fisheries 45 (4): 92-95. [In Chinese].

- Zhang JL, Qin YS (1997) Fishery environment status and management protection countermeasures in Qinghai Lake. Journal of Qinghai Environment 7: 159-163. [In Chinese].
- Zhang RY, Peng ZG, Li GG, Zhang CF, Tang YT, Gan XN, He SP, Zhao K (2013) Ongoing speciation in the Tibetan Plateau *Gymnocypris* species complex. PloS One 8 (8): e71331. https://doi.org/10.1371/journal.pone.0071331
- Zhang YP, Du YY, Lou ZY, Wang T (2013) Molecular phylogeny of schizothoracinae fishes in Gansu Province based on mirochondrial cytochrome *b* gene sequences. Journal of Northwest Normal University (Natural Science) 49 (5): 91-96, 102. https://doi.org/10.16783/j.cnki.nwnuz.2013.05.019
- Zhang Z, Wei J, Chen F, Fang Y, Chang X, Chen J, Huang D, Lei H (2018) Length-weight and length-length relationships of three fish species from the Heihe River, northwest China. Journal of Applied Ichthyology 34 (4): 999-1001. https://doi.org/10.1111/jai.13634
- Zhao GY, Quan JQ, Li LL, Luo ZC, Liu Z (2021) Genetic diversity and phylogenetic relationships among eight populations of *Triplophysa* based on mtDNA Cyt *b* gene sequences. Genomics and Applied Biology 40: 591-598. https://doi.org/10.13417/j.gab.040.000591
- Zhao LH, Wang JL, Zhang YS, Chen Y, Zheng YM, Wang SH (1990) Environment of Keluke Lake and introduction of Valuable fishes. Journal of Fisheries of China 14 (4): 286-296. [In Chinese].
- Zhao L, Chen Y, Zhang Y (1983) Practice and discussion on the introduction and domestication of fishes in Qinghai Plateau. Fisheries Science & Technology Information 5: 17-20. [In Chinese].
- Zhao K, Duan ZY, Peng ZG, Gan XN, Zhang RY, He SP, Zhao XQ (2011) Phylogeography of the endemic *Gymnocypris chilianensis* (Cyprinidae): Sequential westward colonization followed by allopatric evolution in response to cyclical Pleistocene glaciations on the Tibetan Plateau. Molecular Phylogenetics and Evolution 59 (2): 303-310. https://doi.org/10.1016/j.ympev.2011.02.001
- Zhao K, Duan ZY, Peng ZG, Guo SC, Li JB, He SP, Zhao XQ (2009) The youngest split in sympatric schizothoracine fish (Cyprinidae) is shaped by ecological adaptations in a Tibetan Plateau glacier lake. Molecular Ecology 18 (17): 3616-3628. https://doi.org/10.1111/j.1365-294X.2009.04274.x
- Zhao K, Duan ZY, Yang GS, Peng ZG, He SP, Chen YY (2007) Origin of *Gymnocypris przewalskii* and phylogenetic history of *Gymnocypris eckloni* (Teleostei: Cyprinidae). Progress in Natural Science 17: 520-528. https://doi.org/10.1080/10020070708541031
- Zhao K, Li JB, Yang GS, Duan ZY, He SP, Chen YY (2005) Molecular phylogenetics of *Gymnocypris* (Teleostei: Cyprinidae) in Lake Qinghai and adjacent drainages. Chinese Science Bulletin 50 (13): 1325-1333. [In Chinese].
- Zhao K, Yang GS, Li JB, He SP (2006) Phylogenetic structure of *Schizopygopsis pylzovi* populations from mitochondrial cytochrome *b* gene sequence variations. Acta Hydrobiologica Sinica 30 (2): 129-133. [In Chinese].

- Zhao TQ (1984) Taxonomic Problems of some *Nemachilus* fishes. Zoological Research 5 (4): 341-346. [In Chinese].
- Zhao T (1991) Fish-fauna and zoogeographical division of Hexi-Alashan region, the northwest China. Acta Zoologica Sinica 37 (2): 153-167. [In Chinese].
- Zhao TQ, Wang XT (1988) Fishes and fishery problems of Hexi Area, Gansu Province, with descriptions of a new subspecies and an unidentified species of Cobitidae. Journal of Lanzhou University (Natural Sciences) 24 (4): 109-119. [In Chinese].
- Zhao ZM, Liu Y, Lai JS, Zhou J, Zhao H, Ke HY, Huang ZP, Zhang L, Li Q (2022) Length-weight relationships of three fish species from the Shule River in China. Journal of Applied Ichthyology 38 (2): 259-261. https://doi.org/10.1111/jai.14263
- Zhu SQ, Wu YF (1981) A new species and a new subspecies of loaches of the genus *Nemachilus* from Qinghai Province. Acta Zootaxonomica Sinica 6 (2): 221-224. [In Chinese].

(b) Theses

- Lou JM (2019) Species differentiation and ecological adaptation of three Schizothoracinae fish species in Gansu Province. Thesis for the Degree of M.D. of Huazhong Agricultural University, Wuhan. [In Chinese].
- Tang WJ (2008) Genetic diversity of *Schizopygopsis pylzovi* (Teleostei: Cyprinidae) in the Qinghai-Tibetan Plateau. Thesis for the Degree of M.D. of Ocean University of China, Qingdao. [In Chinese].
- Wang T (2019) DNA barcodes and characteristic diagnostic sites of Schizothoracinae fishes from Qinghai Province. Thesis for the Degree of M.D. of Qinghai Normal University, Xining. [In Chinese].
- Zhang F (2013) Phylogeography of *Triplophysa leptosome* in Qilian mountains. Thesis for the Degree of M.D. of Lanzhou University, Lanzhou. [In Chinese].
- Zhang F (2017) Phylogeny and biogeography of *Triplophysa* (Nemacheilinae: Cypriniformes) of the Qilian Mountains area. Thesis for the Degree of Ph.D. of Lanzhou University, Lanzhou. [In Chinese].
- Zhao K (2005) Molecular phylogenetics and phylogeography of Schizothoracinae (Teleostei: Cyprinidae) in Lake Qinghai and adjacent drainages. Thesis for the Degree of Ph.D. of Northwest Agriculture and Forestry Technology University, Xianyang. [In Chinese].

(c) Books

- Bao XK, Zhang LX, Liao JC (2014) Common Vertebrate Atlas in Hexi Corridor. Lanzhou University Press, Lanzhou. [In Chinese]. [ISBN 9787311044886]
- Huang GL (2009) Qinghai Kelukehu-Tuosuhu Wetland Nature Reserve Biodiversity. Beijing Publishing House, Beijing. [In Chinese]. [ISBN 9787200076691]
- Liu YG (2000) Fishery Resources and Regionalization in Gansu province. Lanzhou University Press, Lanzhou. [In Chinese]. [ISBN 7311016940]

- Northwest Plateau Institute of biology, Chinese Academy of Sciences (1989) Qinghai Economic Zoology. Qinghai People's Publishing House, Xining. [In Chinese]. [ISBN 7225002104]
- Qinghai Institute of Biology (1975) Fish Fauna and Biology of Naked Carp in Qinghai Lake. Science Press, Beijing. [In Chinese]. [ISBN 204277415]
- Qinghai Provincial Local Records Compilation Committee (1993) Qinghai Provincial Records of Agriculture and Fisheries. Qinghai People's Publishing House, Xining. [In Chinese]. [ISBN 7225007009]
- Wang JL, Jiang ZQ (1988) Fishery Resources and Fishery Regionalization in Qinghai Province. Qinghai People's Publishing House, Xining. [In Chinese]. [ISBN 7225001531]
- Wang XT (1991) Vertebrate fauna of Gansu. Gansu Science and Technology Press, Lanzhou. [In Chinese]. [ISBN 754240296X]
- Wu YF, Wu CZ (1992) The fishes of the Qinghai-Xizang plateau. Sichuan Publishing House of Science & Technology, Chengdu. [In Chinese]. [ISBN 9787536421684]
- Yellow River Fishery Resources Survey Collaborative Group (1986) Fishery Resources of the Yellow River System. Liaoning Science and Technology Publishing House, Dalian. [In Chinese]. [ISBN 12688·95]
- Zhu SQ (1989) The Loaches of the Subfamily Nemacheilinae in China. Jiangsu Science and Technology Publishing House, Nanjing. [In Chinese]. [ISBN 753450788X]
