# Supplementary figures and images for: Diversity and distribution of fish in the Qilian Mountain Basin
Source: Biodivers Data J. 2022 Aug 12;10:e85992. doi: 10.3897/BDJ.10.e85992 (PMC9848581; doi:10.3897/BDJ.10.e85992)

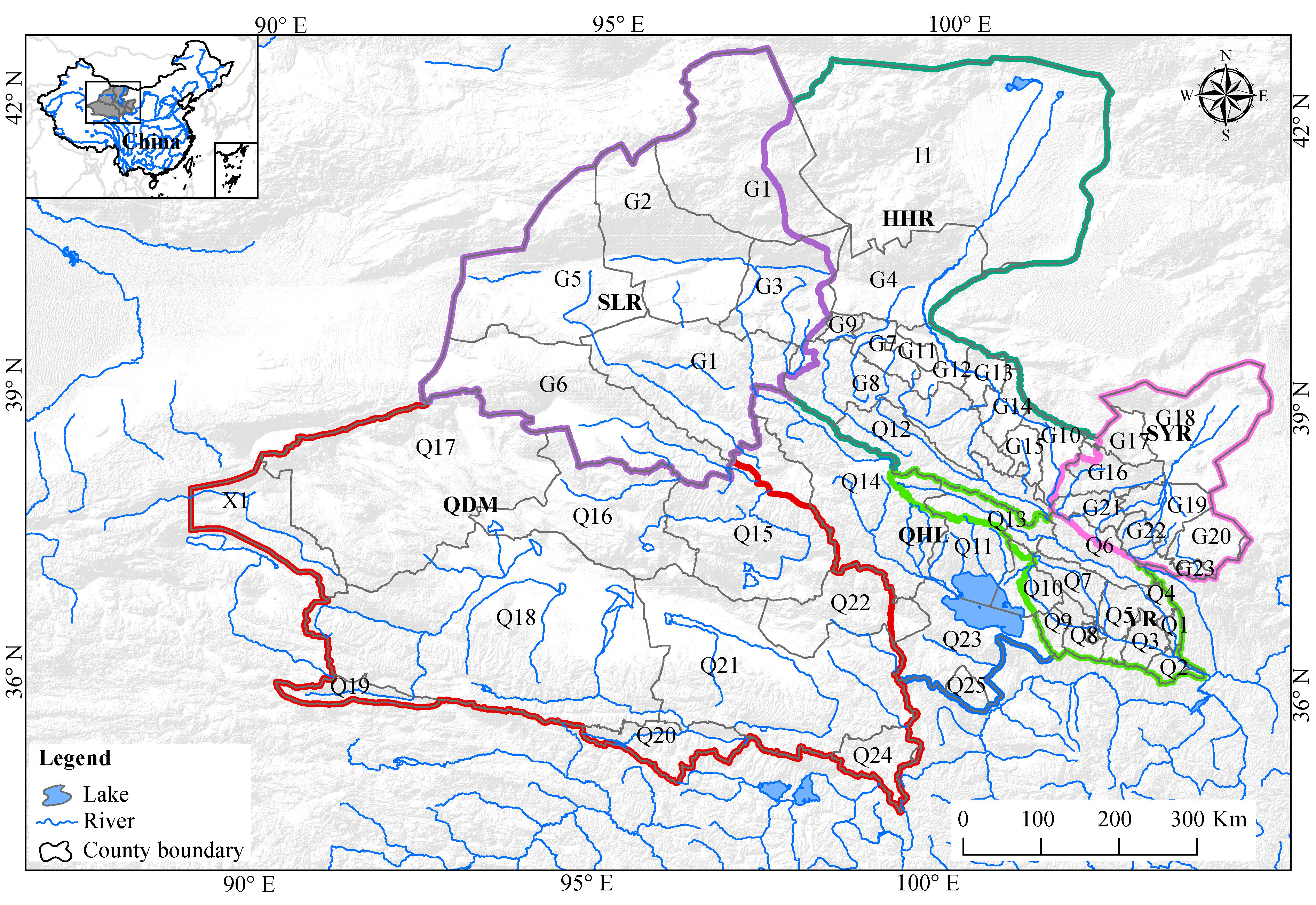

Supplement: Supplementary material 2 — The division of county-level hydrologic units in the Qilian Mountain Basin [file bdj-10-e85992-s002.jpg]

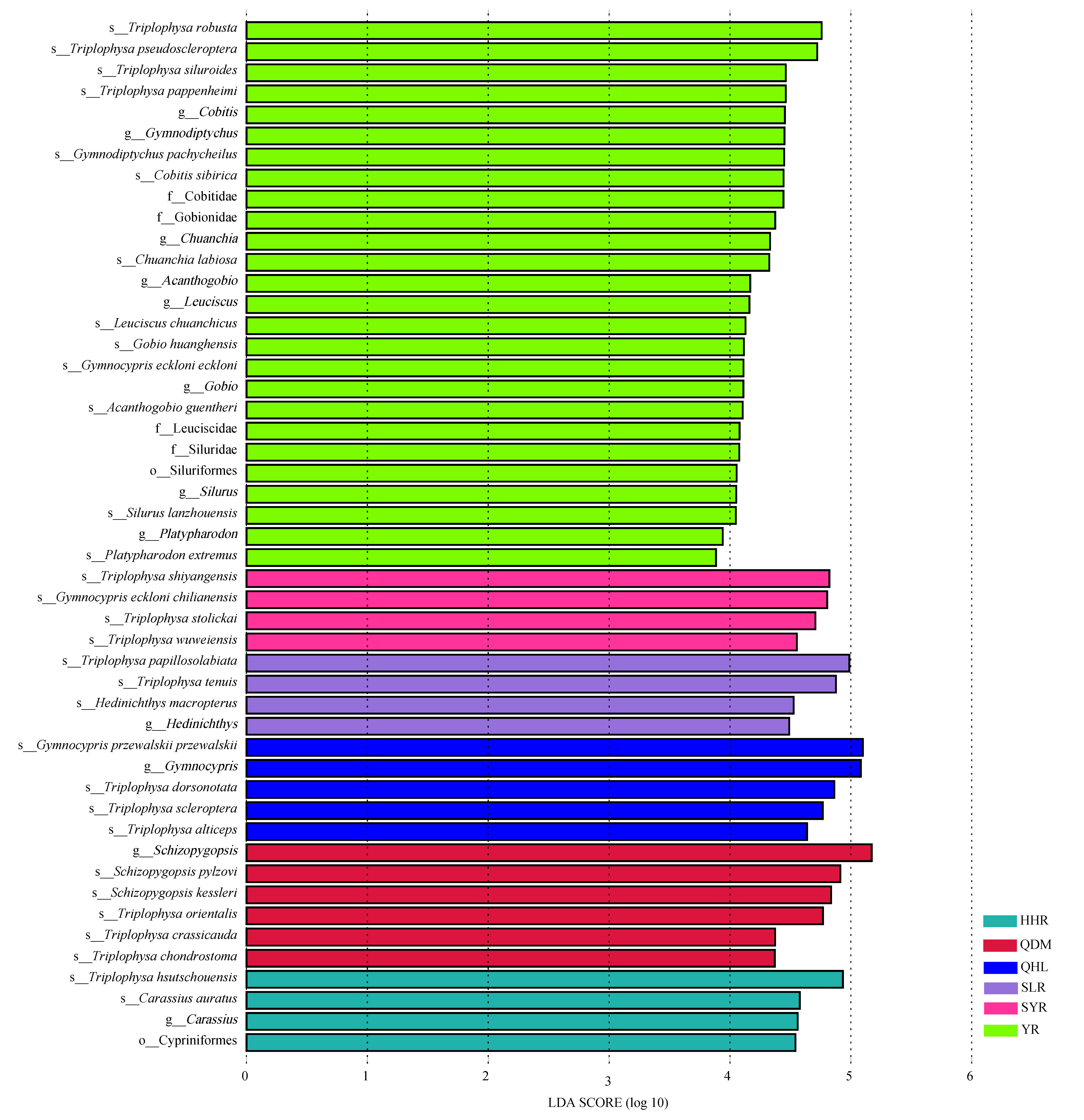

Supplement: Supplementary material 5 — Histogram of the LDA scores computed for features differentially abundant amongst six basins [file bdj-10-e85992-s005.jpg]
