# Supplementary material for: Diversity and distribution of fish in the Qilian Mountain Basin
Source: Biodivers Data J. 2022 Aug 12;10:e85992. doi: 10.3897/BDJ.10.e85992 (PMC9848581; doi:10.3897/BDJ.10.e85992)
Supplement: Supplementary material 3 — The inventory of fish in the Qilian Mountain Basin [file bdj-10-e85992-s003.pdf]

Taxonomic Category				Region					
Orders	Familiies	Genera	Species	HHR	QDM	QHL	SLR	SYR	YR
Anabantiformes	Channidae	*Channa*	*Channa argus*[a]		+				
Beloniformes	Adrianichthyidae	*Oryzias*	*Oryzias latipes*[a]	+					
Cypriniformes	Acheilognathidae	*Rhodeus*	*Rhodeus ocellatus*[a,b]	+					
Cypriniformes	Acheilognathidae	*Rhodeus*	*Rhodeus sinensis*[a,b]						+
Cypriniformes	Cobitidae	*Cobitis*	*Cobitis sibirica*[b]			+		+	+
Cypriniformes	Cobitidae	*Misgurnus*	*Misgurnus anguillicaudatus*[a]	+	+		+	+	+
Cypriniformes	Cobitidae	*Paramisgurnus*	*Paramisgurnus dabryanus*[a,b]	+			+	+	+
Cypriniformes	Cyprinidae	*Carassius*	*Carassius auratus*[c]	+	+		+	+	+
Cypriniformes	Cyprinidae	*Chuanchia*	*Chuanchia labiosa*[b]						+
Cypriniformes	Cyprinidae	*Cyprinus*	*Cyprinus rubrofuscus*[a]	+	+		+	+	+
Cypriniformes	Cyprinidae	*Gymnocypris*	*Gymnocypris eckloni chilianensis*[b]	+			+	+	
Cypriniformes	Cyprinidae	*Gymnocypris*	*Gymnocypris eckloni eckloni*[b]		+				+
Cypriniformes	Cyprinidae	*Gymnocypris*	*Gymnocypris przewalskii ganzihoensis*[b]			+			
Cypriniformes	Cyprinidae	*Gymnocypris*	*Gymnocypris przewalskii przewalskii*[b]		+	+			
Cypriniformes	Cyprinidae	*Gymnodiptychus*	*Gymnodiptychus pachycheilus*[b]						+
Cypriniformes	Cyprinidae	*Platypharodon*	*Platypharodon extremus*[b]						+
Cypriniformes	Cyprinidae	*Schizopygopsis*	*Schizopygopsis kessleri*[b]		+				
Cypriniformes	Cyprinidae	*Schizopygopsis*	*Schizopygopsis pylzovi*[b]	+	+			+	+
Cypriniformes	Gobionidae	*Abbottina*	*Abbottina rivularis*[a]	+	+		+	+	+
Cypriniformes	Gobionidae	*Acanthogobio*	*Acanthogobio guentheri*[b]						+
Cypriniformes	Gobionidae	*Gobio*	*Gobio huanghensis*[b]						+
Cypriniformes	Gobionidae	*Pseudogobio*	*Pseudogobio vaillanti*[a,b]	+					
Cypriniformes	Gobionidae	*Pseudorasbora*	*Pseudorasbora parva*[a]	+	+		+	+	+
Cypriniformes	Leuciscidae	*Leuciscus*	*Leuciscus chuanchicus*[b]						+
Cypriniformes	Leuciscidae	*Rhynchocypris*	*Rhynchocypris lagowskii*[a]	+				+	
Cypriniformes	Nemacheilidae	*Hedinichthys*	*Hedinichthys macropterus*[b]	+			+		

Cypriniformes	Nemacheilidae	*Triplophysa*	*Triplophysa alticeps*[b]		+	+			
Cypriniformes	Nemacheilidae	*Triplophysa*	*Triplophysa bombifrons*[b]				+		
Cypriniformes	Nemacheilidae	*Triplophysa*	*Triplophysa brevicauda*[b]	+	+	+	+		+
Cypriniformes	Nemacheilidae	*Triplophysa*	*Triplophysa cakaensis*[b]			+			
Cypriniformes	Nemacheilidae	*Triplophysa*	*Triplophysa chondrostoma*[b]		+				+
Cypriniformes	Nemacheilidae	*Triplophysa*	*Triplophysa crassicauda*[b]		+				
Cypriniformes	Nemacheilidae	*Triplophysa*	*Triplophysa dorsonotata*	+	+	+	+	+	+
Cypriniformes	Nemacheilidae	*Triplophysa*	*Triplophysa hsutschouensis*[b]	+			+	+	
Cypriniformes	Nemacheilidae	*Triplophysa*	*Triplophysa hutjertjuensis*[b]	+			+	+	
Cypriniformes	Nemacheilidae	*Triplophysa*	*Triplophysa kungessana*[b]		+				+
Cypriniformes	Nemacheilidae	*Triplophysa*	*Triplophysa leptosoma*[b]	+	+	+	+	+	+
Cypriniformes	Nemacheilidae	*Triplophysa*	*Triplophysa microps*		+	+		+	+
Cypriniformes	Nemacheilidae	*Triplophysa*	*Triplophysa orientalis*[b]	+	+		+	+	+
Cypriniformes	Nemacheilidae	*Triplophysa*	*Triplophysa papillosolabiata*[b]	+			+	+	+
Cypriniformes	Nemacheilidae	*Triplophysa*	*Triplophysa pappenheimi*[b]	+					+
Cypriniformes	Nemacheilidae	*Triplophysa*	*Triplophysa pseudoscleroptera*[b]	+	+				+
Cypriniformes	Nemacheilidae	*Triplophysa*	*Triplophysa qilianensis*[b]	+					
Cypriniformes	Nemacheilidae	*Triplophysa*	*Triplophysa robusta*[b]	+			+	+	+
Cypriniformes	Nemacheilidae	*Triplophysa*	*Triplophysa scleroptera*[b]			+			+
Cypriniformes	Nemacheilidae	*Triplophysa*	*Triplophysa shiyangensis*[b]					+	
Cypriniformes	Nemacheilidae	*Triplophysa*	*Triplophysa siluroides*[b]						+
Cypriniformes	Nemacheilidae	*Triplophysa*	*Triplophysa stolickai*	+	+	+	+	+	+
Cypriniformes	Nemacheilidae	*Triplophysa*	*Triplophysa strauchii*	+			+	+	
Cypriniformes	Nemacheilidae	*Triplophysa*	*Triplophysa tenuis*	+			+		
Cypriniformes	Nemacheilidae	*Triplophysa*	*Triplophysa wuweiensis*[b]	+	+		+	+	
Cypriniformes	Xenocyprididae	*Aphyocypris*	*Aphyocypris chinensis*[a]	+			+	+	
Cypriniformes	Xenocyprididae	*Ctenopharyngodon*	*Ctenopharyngodon idella*[a]	+	+		+	+	+
Cypriniformes	Xenocyprididae	*Hemiculter*	*Hemiculter leucisculus*[a]	+	+				+
Cypriniformes	Xenocyprididae	*Hypophthalmichthys*	*Hypophthalmichthys molitrix*[a]	+	+		+	+	+

Cypriniformes	Xenocyprididae	*Hypophthalmichthys*	*Hypophthalmichthys nobilis*[a b]	+	+		+		+
Cypriniformes	Xenocyprididae	*Megalobrama*	*Megalobrama amblycephala*[a b]	+	+				+
Cypriniformes	Xenocyprididae	*Mylopharyngodon*	*Mylopharyngodon piceus*[a]		+				+
Cypriniformes	Xenocyprididae	*Opsariichthys*	*Opsariichthys bidens*[a]	+			+		
Cypriniformes	Xenocyprididae	*Parabramis*	*Parabramis pekinensis*[a]		+				
Cypriniformes	Xenocyprididae	*Zacco*	*Zacco platypus*[a]	+					
Gobiiformes	Gobiidae	*Mugilogobius*	*Mugilogobius myxodermus*[a b]	+			+		
Gobiiformes	Gobiidae	*Rhinogobius*	*Rhinogobius brunneus*[a]	+			+	+	
Gobiiformes	Gobiidae	*Rhinogobius*	*Rhinogobius cliffordpopei*[a b]	+			+	+	+
Gobiiformes	Gobiidae	*Rhinogobius*	*Rhinogobius giurinus*[a]						+
Gobiiformes	Gobiidae	*Rhinogobius*	*Rhinogobius lindbergi*[a b]						+
Gobiiformes	Odontobutidae	*Micropercops*	*Micropercops swinhonis*[a b]	+	+		+		+
Osmeriformes	Osmeridae	*Hypomesus*	*Hypomesus nipponensis*[a]		+				+
Osmeriformes	Osmeridae	*Hypomesus*	*Hypomesus olidus*[a]		+				+
Osmeriformes	Salangidae	*Protosalanx*	*Protosalanx hyalocranius*[a]						+
Salmoniformes	Salmonidae	*Coregonus*	*Coregonus muksun*[a]						+
Salmoniformes	Salmonidae	*Coregonus*	*Coregonus nasus*[a]						+
Salmoniformes	Salmonidae	*Coregonus*	*Coregonus peled*[a]						+
Salmoniformes	Salmonidae	*Oncorhynchus*	*Oncorhynchus mykiss*[a]	+		+		+	+
Siluriformes	Siluridae	*Silurus*	*Silurus asotus*[a]		+			+	
Siluriformes	Siluridae	*Silurus*	*Silurus lanzhouensis*[b]						+
Siluriformes	Ictaluridae	*Ameiurus*	*Ameiurus nebulosus*[a]	+					
Siluriformes	Ictaluridae	*Ictalurus*	*Ictalurus punctatus*[a]	+					
Synbranchiformes	Synbranchidae	*Monopterus*	*Monopterus albus*[a]						+

Basin codes: HHR, Heihe River Basin; QDM, Qaidam Basin; QHL, Qinghai Lake Basin; SLR, Shule River Basin; SYR, Shiyang River Basin; YR, Yellow River Basin. [a] Non-native fish species in the Qilian Mountain Basin. [b] Fish species endemic to China. [C] *Carassius auratus* is an indigenous species in the HHR, SLR and SYR but a non-native species in the QDM, QHL and YR.
