# Supplementary material for: Diversity and distribution of fish in the Qilian Mountain Basin
Source: Biodivers Data J. 2022 Aug 12;10:e85992. doi: 10.3897/BDJ.10.e85992 (PMC9848581; doi:10.3897/BDJ.10.e85992)
Supplement: Supplementary material 4 — Conservation status of native fish species in the Qilian Mountain Basin [file bdj-10-e85992-s004.pdf]

Species	Protection class		Endangered categories
	National level	Provincial level	
Cobitis sibirica			LC
Carassius auratus			LC
Chuanchia labiosa	II	Q	EN
Gymnocypris eckloni chilianensis		G	
Gymnocypris eckloni eckloni		G	VU
Gymnocypris przewalskii ganzihoensis		Q	
Gymnocypris przewalskii przewalskii		Q	VU
Gymnodiptychus pachycheilus	II	G	VU
Platypharodon extremus	II	G; Q	EN
Schizopygopsis kessleri			DD
Schizopygopsis pylzovi		G	VU
Acanthogobio guentheri			EN
Gobio huanghensis			EN
Leuciscus chuanchicus		G; Q	CR
Hedinichthys macropterus			VU
Triplophysa alticeps			NT
Triplophysa bombifrons			CR
Triplophysa brevicauda			DD
Triplophysa cakaensis		Q	EX
Triplophysa chondrostoma			DD
Triplophysa crassicauda			DD
Triplophysa dorsonotata			DD
Triplophysa hsutschouensis			DD
Triplophysa hutjertjuensis			VU
Triplophysa kungessana			DD
Triplophysa leptosoma			LC
Triplophysa microps			DD
Triplophysa orientalis			LC
Triplophysa papillosolabiata			DD
Triplophysa pappenheimi		G	EN
Triplophysa pseudoscleroptera			DD
Triplophysa qilianensis			
Triplophysa robusta			LC
Triplophysa scleroptera			LC
Triplophysa shiyangensis			DD
Triplophysa siluroides	II	G; Q	VU
Triplophysa stolickai			LC
Triplophysa strauchii			DD
Triplophysa tenuis			DD
Triplophysa wuweiensis			DD
Silurus lanzhouensis		G; Q	EN

Protection class: II, National second class protected animal; G, Key protected wild animals in Gansu Province; Q, Key protected aquatic wildlife in Qinghai Province. Endangered categories: DD, Data

Deficient; LC, Least Concern; NT, Near Threatened; VU, Vulnerable; EN, Endangered; CR, Critically Endangered; EX, Extinct.
